# Implementation of Principal Component Analysis (PCA)/Singular Value Decomposition (SVD) and Neural Networks in Constructing a Reduced-Order Model for Virtual Sensing of Mechanical Stress

**DOI:** 10.3390/s24248065

**Published:** 2024-12-18

**Authors:** M. A. Melgarejo, A. Pérez, D. Ruiz, A. Casas, F. González, V. González de Lena Alonso

**Affiliations:** Fundación Centro Tecnológico CTC—Scientific and Technological Park of Cantabria (PCTCAN), Street Isabel Torres Nº 1, 39011 Santander, Spain

**Keywords:** predictive maintenance, virtual sensor, reduced-order model (ROM), PCA, neural networks

## Abstract

This study presents the design and validation of a numerical method based on an AI-driven ROM framework for implementing stress virtual sensing. By leveraging Reduced-Order Models (ROMs), the research aims to develop a virtual stress transducer capable of the real-time monitoring of mechanical stresses in mechanical components previously analyzed with high-resolution FEM simulations under a wide range of multiple load scenarios. The ROM is constructed through neural networks trained on Finite Element Method (FEM) outputs from multiple scenarios, resulting in a simplified yet highly accurate model that can be easily implemented digitally. The ANN model achieves a prediction error of ${\mathrm{MAE}}_{\mathrm{test}}=\left(0.04\pm 0.06\right)\;\mathrm{MPa}$ for the instantaneous mechanical stress predictions, evaluated over the entire range of stress values (0 to 5.32 MPa) across the component structure. The virtual sensor is capable of producing a quasi-instantaneous, detailed full stress map of the component in just 0.13 s using the ROM, for any combination of 4-load inputs, compared to the 6 min and 31 s required by the FEM. Thus, the approach significantly reduces computational complexity while maintaining a high degree of precision, enabling efficient real-time monitoring. The proposed method’s effectiveness is demonstrated through rigorous ROM validation, underscoring its potential for stress control. This precise AI-driven procedure opens new horizons for predictive maintenance strategies centered on stress cycle monitoring.

## 1. Introduction

The use of digital AI tools to enhance and optimize manufacturing has significantly benefited the entire industry over the past few decades. Through virtually representing factories, resources, workforce, etc., digital manufacturing builds models and simulates products and process developments [1,2,3,4,5]. The progress in information and communication technologies has promoted the development of manufacturing greatly [6,7]. Computer-aided technologies are developing quickly and playing an increasingly critical and typical role in industry [8,9,10]. Big data [11,12], Internet of Things (IoT) [13], Artificial Intelligence (AI) and Machine Learning (ML), and cloud computing and new sensors [14], among others, are developing rapidly and show big potential in every aspect of Industry 4.0 [15,16,17,18]. All these technologies provide opportunities for the integration of the physical and digital worlds, which is an inevitable trend to address.

Virtual Sensing is also emerging as a key concept to implement all of these technologies aimed to support real-time process monitoring, adaptive control, and process optimization, which are crucial components when creating accurate digital assets. So this concept is emerging as a promising tool in the Industry 4.0 landscape. Ref. [19] discusses the trends and challenges of virtual sensing technology, also known as soft sensors, in process industries, focusing on improving product quality and operational efficiency. It highlights key advantages over traditional hardware sensors, such as reduced maintenance needs, improved accuracy, and the ability to estimate variables that are hard to measure in real-time. Some authors [20] have highlighted how virtual sensors and technologies can reduce costs in industry compared to physical devices. Furthermore, a recent study [21] reveals that virtual sensing can detect loading time series in extreme offshore floating platform scenarios, surpassing expensive physical sensors by enabling failure prediction and preventing downtime due to structural issues. Cristaldi et al. [22] remark how virtual sensors can improve the reliability and availability of the application domain while avoiding the need for extra hardware complexity. In the area of corrosion, a recent work [23] explores the use of advanced ML methods to predict corrosion in industrial cooling water pipelines, ultimately leading to the creation of a virtual corrosion sensor that relies on process variables, such as pH and temperature.

On the other hand, the Reduced-Order Model (ROM) is a computational tool widely used in industry to simplify some high-order systems, i.e., systems with many variables or degrees of freedom. In this regard, some techniques like compressing the full-order Ordinary Differential Equations (ODEs) to facilitate faster computations and reduce storage requirements, while preserving the essential characteristics of the original system, have been proposed [24]. Nevertheless, the recent emergence of AI as an enabling tool for advanced mathematical modeling, has enabled the application of new techniques, in which differential equations can be replaced by supervised and unsupervised learning-based approaches. Therefore, AI enables the synthesis of large-scale modeling scenarios with manageable computational requirements [25]. Well-established algorithms such as genetic algorithms [26], ANNs[27], fuzzy logic [28,29], particle swarm optimization [30], and simulated annealing have shown promise in solving ROM problems, so techniques can aid in automating the reduction process, optimizing system performance, and handling complex datasets efficiently.

Nonetheless, to further reduce the complexity of systems, the Principal Component Analysis (PCA) technique can be also applied. It identifies the directions of maximum variance and and enables the projection of the high dimensional data onto them, thereby reducing the number of features while preserving most of variance of the original data. Therefore, PCA facilitates the reduction process in ROM [31], and contributes to creating more effective Reduced-Order Models by identifying dominant components of the numerical space obtained from simulations. Other similar procedures that could be employed in a ROM, instead of PCA, are truncated balanced realization [32], Hankel-norm reduction [33], proper orthogonal decomposition (POD), the T-SNE algorithm [34], and Krylov subspace-based methods. Nevertheless, PCA is conceptually simpler than some methods, such as truncated balanced realization, and computationally faster than others, such as T-SNE.

Recent works in the literature have focused on combining virtual sensing with ROMs to enhance real-time simulation efficiency and accuracy in engineering applications, where complex, data-intensive tasks and real-time predictions are critical for system performance. Typically, performance assessment relies on complex simulations. In particular, for those scenarios involving mechanical, structural, fluid, and thermal physics, the Finite Element Method (FEM) is commonly employed. This computational technique enables the modeling of a system’s physical behavior by dividing it into small elements and delivers highly detailed results. Nevertheless, as FEM models are often complex and computationally intensive, especially for real-time applications, model reduction techniques are applied to retain the essential characteristics of the FEM model, enabling real-time performance with relatively high accuracy. This approach is exemplified in works such as [35], where an Autoencoder State Estimation (AE-SE) framework is trained with these kinds of numerical simulations, integrating sparse and noisy sensor data into a ML-based ROM. This method achieves a 70% improvement in reconstruction efficiency while preserving key physical characteristics in unsteady flow fields. Furthermore, in [36], a virtual sensor that estimates real-time cutting edge temperatures in machining processes using temperature measurements is presented. This ROM-based state space model, created using the Krylov subspace method, enables accurate real-time heat transfer analysis. Additionally, in the context of microfluidic devices, ref. [37] proposes a virtual sensor that combines CAE simulations with model order reduction (MOR) techniques to predict fluid interface positions in real-time, based on physically sensed input data. In this case, the reduction method is developed using a Canonical Polyadic Decomposition (CPD) of tensors.

On the other hand, in the field of mechanical or structural analysis, studies such as [38,39] introduce structural monitoring Reduced-Order Models (ROMs) for floating offshore wind turbines (FOWTs). These models rely on time-domain load inputs and select principal component models by the PCA or POD method to linearly combine them, enabling the study of time-domain responses under specific design load cases. A similar approach is adopted in [40] for simplified mechanical FEMs with thousands of nodes, where short time-domain responses are reconstructed using Principal Component Analysis methodology, with ROM training based on the Empirical Cubature Method (ECM). In addition, ref. [41] applies a similar technique with a 2D FEM model, containing only hundreds of nodes. However, none of these works address the challenges of high-order mesh definitions in complex mechanical parts under multiple load inputs.

Indeed, there are no clear references that scale PCA-ROM-based solutions for virtual sensors intended to monitor mechanical stress in complex parts. This is particularly true for industrial components subjected to a linear loading regime and represented by finely meshed FEM models [42]. This applies to pressure vessel components designed under the ASME BPVC, structural elements governed by Eurocode, and various parts designed according to ANSI/ASCE standards. Furthermore, these models require simulations across a broad range of scenarios generated by multiple load inputs that must be carefully combined to cover the component’s entire operational range. Such an approach could produce an overwhelming volume of snapshot data, making it computationally infeasible to process, even with high-performance computing resources [43]. Additionally, a mechanical virtual sensor functions as an iterative system with a large number of outputs—one for each resultant nodal stress value—and relatively few load/force inputs [44]. This imbalance presents significant challenges in constructing accurate Reduced-Order Models for this case.

To address these challenges, a novel, robust, and scalable method is proposed in this work to train a high-accuracy, low-computation virtual sensor for mechanical components operating under a linear loading regime, independent of the initial model’s mesh refinement or the complexity of the load input framework. This method incorporates techniques for processing high-dimensional data that would be otherwise intractable with conventional tools and also leverages AI to train the core ROM, enabling it to capture and learn the nodal interrelationships within a geometrically complex component.

### Structure of the Document

This work is divided into four main sections. Section 2 describes the methodology employed, focusing on the FEM technique, as well as the conceptual framework and the mathematical principles of the ROM. Section 3 provides details about the implementation of the methodology, including data preparation and the specific configuration of the AI model. Section 4 discusses the obtained results. Section 5 presents the main conclusions drawn, as well as the future developments.

## 2. Methodology

The general procedure began with the simulation of the structure, followed by the application of FEM to calculate the stresses generated in this structure when a load was applied to its top. Subsequently, FEM data were used to train a Deep Learning (DL) algorithm capable of predicting stresses in the structure for new loads.

A more detailed breakdown of the steps is as follows:

- Structural design: The first step involved designing the structure itself, defining the geometry, materials, and boundary conditions such as restraints and applied loads.
- FEM: Equilibrium equations were solved for each finite element, considering the applied loads and boundary conditions, providing detailed insights into stress distribution throughout the structure.
- Data preprocessing: Given the large volume of output data generated from multiple load combination scenarios simulated with FEM, the data were processed in batches. PCA was applied as a data reduction technique, ensuring that the dataset was manageable for subsequent training steps.
- DL training: A ROM was created to learn the complex relationships between applied load and resulting stresses. Once the model was trained, validation was performed to evaluate its performance.

### 2.1. General Description of the FEM

**Figure 1:**
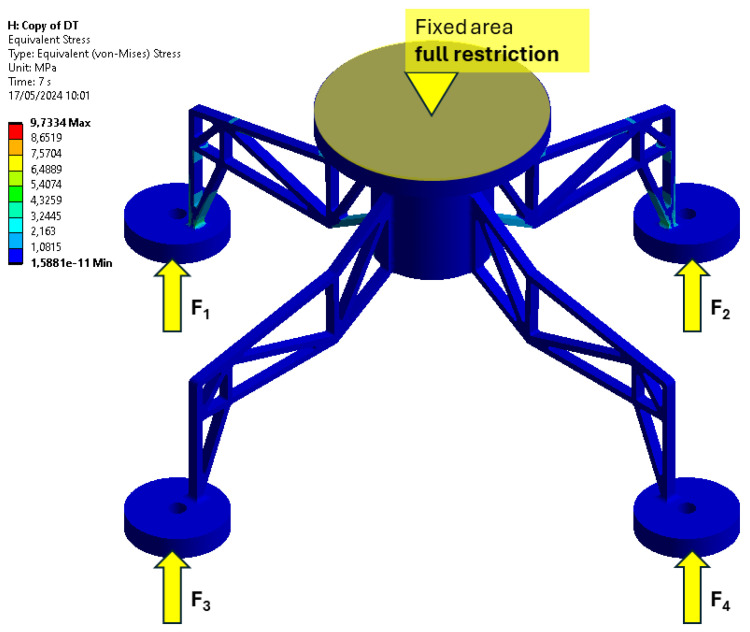
FEM model with representation of the equivalent stress (von Mises) analysis on a support structure. The shaded areas indicate regions of higher stress, with a full restriction at the central upper part. The applied forces $F_{1}$, $F_{2}$, $F_{3}$, and $F_{4}$ are represented by yellow arrows.

The mechanical model proposed in this work is a four-legged support structure designed to represent an industrial element under mechanical stress. The model, represented in Figure 1, consists of a central cylindrical section with four legs extending outwards in a symmetrical arrangement. Each leg ends in a circular base, which serves as a receptor of force application.

As indicated in Figure 1, the top face of the central cylindrical section is marked as a fixed area with full restriction. This constraint ensures that the top face is completely constrained, meaning it cannot move or rotate in any direction, thereby maintaining the structure’s stability while the applied forces act on it. Four vertical upward forces, labeled as $F_{1}$, $F_{2}$, $F_{3}$, and $F_{4}$, are applied at the bases of the four legs. These forces are depicted as yellow arrows pointing upward, representing the directions in which the forces act.

A FEM model of the structure is defined as a baseline to obtain data for training the further AI-based ROM. The FEM software of choice is ANSYS Mechanical [45]. The mesh of the model is composed of a total of 141,100 nodes and 83,091 SOLID187 [46] elements. SOLID187 is a 3-dimensional high-order element defined by 10 nodes with three degrees of freedom at each node. This allows for a detailed and accurate representation of the structure’s mechanical behavior under various loading conditions.

**Figure 2:**
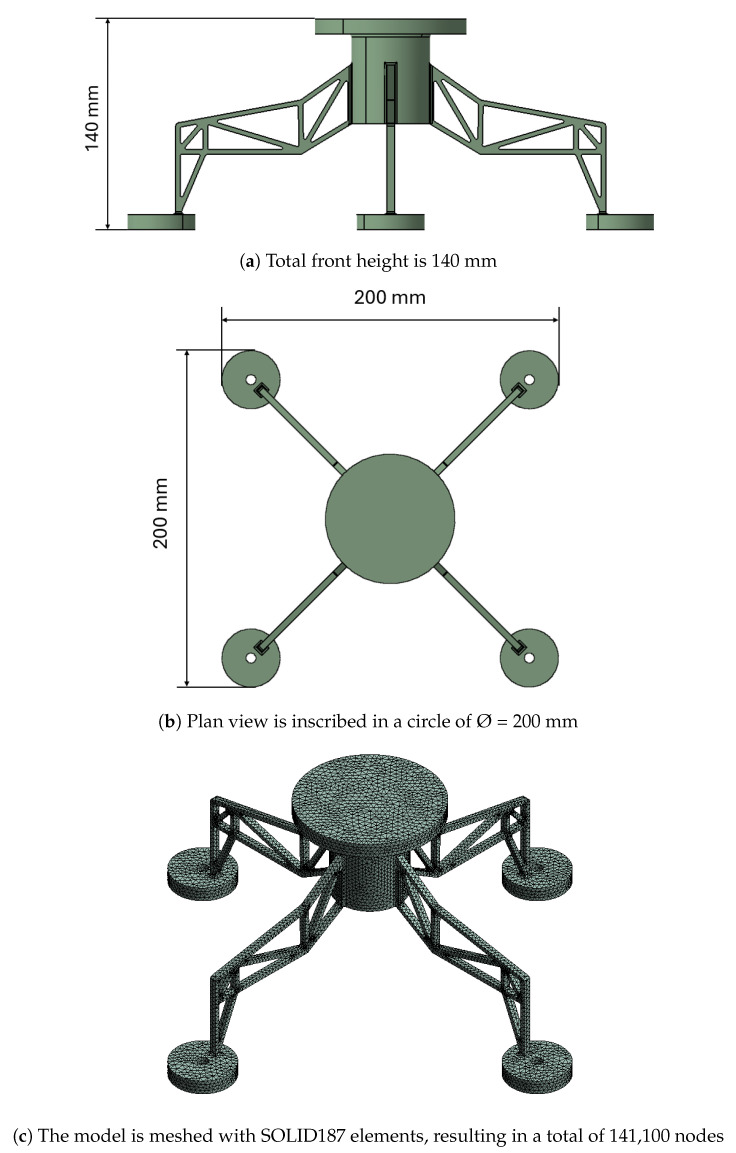
Representation of the FEM model in different views: front, plan, and 3D isometric.

In Figure 2, another representation of the model is shown. Its total height projected from the front is 140 mm as depicted in the top image, while its plan view is inscribed in a circle with a diameter of 200 mm as shown in the central image. The bottom image displays the model projected in a 3D isometric view, where the mesh is drawn.

This model is used to analyze the structural integrity and mechanical behavior of the support structure under the given loading conditions. The von Mises stress ($\sigma_{v}$) is calculated for each of the 141,100 nodes. $\sigma_{v}$ is calculated for each node using the principal stresses, $\sigma_{1}$, $\sigma_{2}$, and $\sigma_{3}$, and is given by Equation (1). These are the maximum, intermediate, and minimum normal stresses at each node, respectively:

(1) $$\sigma_{v}=\sqrt{\frac{{\left(\sigma_{1}-\sigma_{2}\right)}^{2}+{\left(\sigma_{2}-\sigma_{3}\right)}^{2}+{\left(\sigma_{3}-\sigma_{1}\right)}^{2}}{2}}$$

The simulation is launched for a specific combination of loads $F_{1}$, $F_{2}$, $F_{3}$, and $F_{4}$, constituting a single scenario. The ANSYS model generates a text file for this scenario containing detailed information about the structural response. Specifically, the file includes the Equivalent von Mises Stress $\sigma_{v}$ for each of the 141,100 nodes in the model, along with the corresponding node IDs and their XYZ spatial positions.

### 2.2. Theoretical Foundations of the ROM

The ROM consists of two main components: a space or dimensional reduction and an DL model. The former simplifies the problem without significant information loss and enables the swift training of the latter, which is capable of predicting the outcomes of new cases.

Dimensionality reduction techniques are commonly employed to increase the performance of ML models when the number of input variables is high compared to the number of output variables. Typically, there is one or only a few target variables; however, in this study, the opposite is true. Here, the input space, representing the forces applied to the supports of the structure described in Figure 1, is 4-dimensional, as each input scenario is defined by the vector in Equation (2). In contrast, the output space, constituted by the von Mises stress at each individual finite element, has a much higher dimension. The number of target variables corresponds to the number of finite elements (*m*; see Equation (3)). Subsequently, the mathematical formulation of spatial reduction for this use case is presented.

Let *f* be the force applied to the structure (a single scenario),

(2) $$f=[F_{1},F_{2},F_{3},F_{4}]$$

where each $F_{i}$ corresponds to the force on each respective support, and $S_{j}$ represents the von Mises stress resulting for each of the *m* finite elements in the simulation:

(3) $$s=[S_{1},S_{2},\ldots ,S_{m}]$$

If we consider all simulation scenarios, these pairs f−s can be arranged in the form of the following matrices F and S (see Equation (4)):

(4) $$F=\left[\begin{array}{cccc} F_{1,1} & F_{1,2} & F_{1,3} & F_{1,4}\\ F_{2,1} & F_{2,2} & F_{2,3} & F_{2,4}\\ \vdots & \vdots & \ddots & \vdots \\ F_{n,1} & F_{n,2} & F_{n,3} & F_{n,4} \end{array}\right]\;;\;S=\left[\begin{array}{cccc} S_{1,1} & S_{1,2} & \cdots & S_{1,m}\\ S_{2,1} & S_{2,2} & \cdots & S_{2,m}\\ \vdots & \vdots & \ddots & \vdots \\ S_{n,1} & S_{n,2} & \cdots & S_{n,m} \end{array}\right]$$

with *n* as the number of scenarios and *m* = 141,100 as the number of nodes.

Since any modification to F results in a change in S, the ROM represents the function F that relates the inputs and outputs (see Equation (5)):

(5) F(F)≈S

#### 2.2.1. Numerical Reduction with PCA

**Figure 3:**
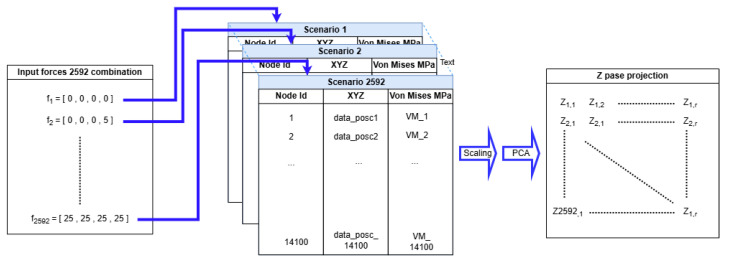
Scheme showing the implementation of 2592 combinations of input forces for the 4 legs of the FEM model using ANSYS Mechanical. Each combination generates a stress scenario for all 141,100 nodes of the model. The von Mises stress information is scaled and processed together using PCA based on SVD truncation to obtain a highly reduced and manageable training dataset for a DL-based model.

Given the nature of the model, it represents the best possible approximation to reality, making a scenario where f(F)=S is neither achievable nor desirable. The spatial reduction here consists of transforming S such that the output dimensions are drastically reduced, yet with minimal loss of information. This can be achieved by applying the PCA technique [47], which consists of finding a projection of S in a lower-dimension space P that preserves the main part of its variance as illustrated in Figure 3.

The projection method implemented in PCA, known as Singular Value Decomposition (SVD), is a generalization of the eigen-decomposition, applicable to rectangular matrices [48]. According to SVD, the normalized and centered_version of $S_{n\times m}$, which is named ${S^{\prime }}_{n\times m}$, can be expressed as the product represented in Equation (6):

(6) $${S^{\prime }}_{n\times m}=U_{n\times n}\Sigma_{n\times m}V_{m\times m}^{T}$$

where $\Sigma_{n\times m}$ is a diagonal matrix that comprises the singular values of $S^{\prime }$, and $U_{n\times n}$ and $V_{m\times m}^{T}$ are squared matrices composed by the singular vectors of $S^{\prime }{S^{\prime }}^{T}$ and ${S^{\prime }}^{T}S^{\prime }$ as columns, respectively. In this study, singular values and vectors belong to the covariance matrix of a subset $S_{n^{\prime }\times m}$, where n’ < n.

Dimensionality reduction can be performed by truncating the matrix $\Sigma_{n\times m}$, i.e., by removing columns from right to left and rows from bottom to top, to the same extent. Consequently, the dimensions of $U_{n\times n}$ and $V_{m\times m}^{T}$ are modified, resulting in a lower-dimensional approximation of $S^{\prime }$ as expressed in Equation (7):

(7) $${S^{\prime }}_{n\times m}\approx U_{n\times r}\Sigma_{r\times r}V_{r\times m}^{T}$$

where *r* is the reduced rank in the least squares sense. The entire value *r* is established by assessing the inequality of Equation (8). According to this criterion, the truncation order *r* is chosen such that the truncation error (${\mathrm{error}}_{\mathrm{trunc}}$) can be less than 1%. This ensures that the reduced representation retains significant fidelity while achieving a sensible reduction in the original space:

(8) $${\mathrm{error}}_{\mathrm{trunc}}=\frac{\frac{1}{nxm}\sum_{i=1}^{nxm}{∥x_{\mathrm{real}}^{i}-x_{\mathrm{aprox}}^{i}∥}^{2}}{\frac{1}{nxm}\sum_{i=1}^{nxm}{∥x_{\mathrm{real}}^{i}∥}^{2}}\cdot 100\leq 1\%$$

where $x^{i}$ represent any of the nxm values of the matrices S.

In the context of dimensional reduction for DL models, training with the projection matrix $Z_{n\times r}$ (see Equation (9)) offers several advantages:

(9) $$Z_{n\times r}={S^{\prime }}_{n\times m}V_{m\times r}=U_{n\times r}\Sigma_{r\times r}$$

Apart from significant dimensional reduction, which reduces the number of outputs in the training model, the method of projecting $Z_{n\times r}$ captures the most relevant information for prediction, optimizing the representation of the output data. This approach maximizes the significance of features in predicting outcomes, potentially leading to enhanced model performance. Moreover, this technique represents a transformation of the data that maximizes the variability in directions most relevant for prediction. Thus, $Z_{n\times r}$ (see Equation (9)) will be used as the output to be implemented by the further ML model.

#### 2.2.2. Dataset Splitting Strategy in PCA

It is crucial to consider the role of dataset splitting in the application of PCA. While dataset splitting is a standard practice in PCA, it is often less discussed or emphasized compared to its application in DL models. In PCA, the principal components are computed exclusively using the training set to define the new feature space. This ensures that the PCA transformation is based solely on the data used during training, avoiding any influence from the validation or test datasets.

It is also essential that the dataset partitions used for PCA and the downstream DL model remain identical. This consistency ensures that the validation and test datasets are projected into the same principal component space defined by the training data, maintaining coherence between the dimensional reduction and the DL processes. Without this alignment, inconsistencies could arise that compromise the validity of model evaluation and comparisons between training and testing phases. Properly aligning the partitions is therefore critical to ensuring the integrity of the overall workflow.

#### 2.2.3. Deep Learning Algorithm

**Justification for Using the ANN**

During the initial phase of our study, several Deep- and Machine Learning models were evaluated to address the challenge of mapping a low-dimensional input vector with four values to a significantly larger output vector with r values through a supervised learning process. The models considered included Singular Value Decomposition Regression (SVDR), Random Forest (RF) regression, and eXtreme Gradient Boosting (XGBOOST). However, the Artificial Neural Network (ANN) proved to be the most effective solution due to the following:

- Capturing complex and spatial dependencies;
- Adaptability to high-dimensional output spaces;
- Capacity for hierarchical feature learning.

This selection is further justified by the intricate nature of the problem. Unlike typical AI modeling tasks, where input features outnumber output labels, this case presents the opposite scenario: even after dimensional reduction, the output retains a substantial number of elements compared to the simpler input. This unique challenge underscores the importance of ANNs, which excel at processing high-dimensional data and uncovering complex relational structures. Their layered architecture and interconnected nodes provide a flexible and robust framework capable of addressing such disparities effectively.

**Mechanisms of ANNs**

In ANNs, the mechanisms of feedforward and backpropagation are crucial for training. Feedforward involves data moving from the input layer to the output layer, passing through several hidden layers. Each neuron in these layers processes the input using assigned weights and a non-linear activation function, culminating in the network’s predictions. Backpropagation complements this by adjusting the weights based on the error between the network’s predictions and the actual target values. This error is propagated back through the network, guiding the optimization of weights through algorithms like gradient descent to reduce future prediction errors. These mechanisms effectively address high-dimensional outputs. Feedforward handles complex mappings from input to output, while backpropagation minimizes errors across the output matrix, ensuring accurate predictions despite the output’s complexity.

**ANN Model Framework**

This section outlines the theoretical framework and key principles underlying the ANN architecture used in this study. The focus is on describing the general structure and essential components that guide the implementation of the neural network. Specific implementation details, such as exact parameter values and experimental configurations, are provided in the subsequent sections (refer to Section 3.3). By separating the theoretical design from the practical details, this section ensures a clear understanding of the conceptual foundation of the methodology.

The ANN model employed in this approach is designed to address the complex relationship between input and output variables in a structured and efficient manner.

- Key definitions: the architecture employs sequential dense layers in a feedforward configuration, where the output of one layer serves as the input for the next. This setup progressively transforms raw input features into meaningful outputs while maintaining computational efficiency and reliability. The dense layers, which form the core of the model, are introduced through the following key definitions:
  – Dense layers: fully connected layers that enable the network to learn intricate patterns by connecting each neuron to all neurons in the preceding layer.
  – Activation function: all dense layers, except the output layer, employ the *ReLU* (Rectified Linear Unit) activation function to introduce non-linearity, facilitating the modeling of complex relationships.
  – Neuron scaling: the number of neurons in each dense layer is scaled relative to the number of input features, balancing model complexity with computational feasibility.
- The overall structure and functionality of the ANN architecture are outlined below:
  – Input layer: a dense layer that directly receives the four input features ($F_{1},F_{2},F_{3},F_{4}$), initialized with constant weights and employing the *ReLU* activation function to capture non-linear input–output relationships from the outset.
  – Hidden layers: intermediate layers positioned between the input and output layers, responsible for processing input features into abstract representations through dense connections and activation functions.
  – Output layer: a dense layer that maps the learned features to the high-dimensional output space (*r* outputs). This layer, suited for regression tasks, does not use an activation function, allowing direct numerical predictions.
- Model compilation: after defining the ANN architecture, the model must be compiled with specific settings that define how it learns and how its performance is assessed. This involves selecting an optimizer to adjust the model’s parameters, defining a loss function to quantify prediction errors, and establishing evaluation metrics to measure the model’s effectiveness in capturing the relationships between input and output variables. These elements, detailed below, are fundamental to ensuring that the training process is both efficient and capable of producing accurate predictions.
  – Optimizer: the Adam optimizer is selected for its efficiency in managing sparse gradients and adapting learning rates, facilitating better and faster convergence.
  – Loss Function: the ANN uses the Mean Squared (MSE) as the loss function, which is well suited for regression tasks aiming to minimize error. MSE is a common loss function used for regression tasks which measures the average squared difference between the predicted and actual values. The formula for MSE is given by Equation (10):
    (10) $$\mathrm{MSE}=\frac{1}{n}\sum_{i=1}^{n}{\left(Z_{i,j}-{\hat{Z}}_{i,j}\right)}^{2}$$
    where we have the following:
    ∗ *n* is the number of samples.
    ∗ $Z_{i,j}$ is the actual value for the (i,j)-th sample.
    ∗ ${\hat{Z}}_{i,j}$ is the predicted value for the (i,j)-th sample.
  – Early Stopping: it is a regularization technique employed to halt the training process when the model’s performance on the validation set ceases to improve. Specifically, if the validation loss does not decrease after a predefined number of consecutive epochs, the training is stopped. This prevents overfitting by avoiding unnecessary iterations, conserving computational resources, and ensuring the model retains its generalization capabilities.
  – Performance Evaluation: the performance of the model is assessed using metrics such as the MSE, the Mean Absolute Error (MAE), and the Pearson correlation coefficient. The formula for MAE is given by Equation (11):
    (11) $$\mathrm{MAE}=\frac{1}{n}\sum_{i=1}^{n}\left|Z_{i,j}-{\hat{Z}}_{i,j}\right|$$
    where *n*, $Z_{i,j}$, and ${\hat{Z}}_{i,j}$ have the same meanings as in the MSE formula.
    The Pearson correlation coefficient is a statistical measure that quantifies the linear relationship between two variables, and it is given by Equation (12):
    (12) $$r=\frac{\sum_{i=1}^{n}\left(X_{i}-\bar{X}\right)\left(Y_{i}-\bar{Y}\right)}{\sqrt{\sum_{i=1}^{n}{\left(X_{i}-\bar{X}\right)}^{2}}\sqrt{\sum_{i=1}^{n}{\left(Y_{i}-\bar{Y}\right)}^{2}}}$$
    where we have the following:
    ∗ $X_{i}$ and $Y_{i}$ are individual data points of variables *X* and *Y*.
    ∗ $\bar{X}$ and $\bar{Y}$ are the means of *X* and *Y*, respectively.
    ∗ *n* denotes the number of observations.
    Its value ranges from −1 to 1, where the following hold:
    ∗ 1 indicates a perfect positive correlation, meaning that as one variable increases, the other increases proportionally.
    ∗ −1 indicates a perfect negative correlation, meaning that as one variable increases, the other decreases proportionally.
    ∗ 0 indicates no linear relationship between the two variables.

**ANN Training and Validation Approach**

To effectively implement the ANN model, it is crucial to have a robust and representative dataset that captures the complex relationships between the input features and the projected outputs.

To achieve this, the dataset was split into training, validation, and test sets, using the same partitions as those applied in the PCA techniques mentioned in Section 2.2.2. The majority of the data points were allocated to the training set to ensure the model was exposed to a wide range of scenarios during training. A smaller portion was reserved for validation and testing, enabling an unbiased evaluation of the model’s performance. Prior to proceeding with these partitions, a thorough analysis was conducted to ensure their quality and representativeness.

Furthermore, the training involves the implementation of numerous iteration epochs with a specified batch size. This means that the network is trained with smaller groups of observations (batch) each time, until all the observations in the dataset have been used to update the model. This process is repeated multiple times, across several epochs. Generally, more epochs improve model performance, but too many can lead to overfitting. Therefore, this issue is detected and mitigated by monitoring the evolution of the metrics during the training and validation processes together, selecting the best hyperparameters for the model, including epochs and batch size, as well as those that define the architecture of the model, defined.

#### 2.2.4. PCA Inversion and Descaling

The aim now here is to show how to obtain a new calculated von Mises stress response for all the *m* nodes of the FEM model by using the ROM. The $S_{1\times m}$ von Mises response through the FEM ANSYS model for a given random input vector $f=[F_{1},F_{2},F_{3},F_{4}]$ such that $F_{i}\in \left[0,25\right]$ N, which is not contained in any of the training scenarios. It must be noted that the dimensions are 1×m because the force input combination is just the selected one.

Thanks to the already trained DL model as described in Section 2.2.3, the von Mises response of f in the reduced space can be predicted ($Z_{\mathrm{predict}|1\times r}$). Then, this response can be projected back to the original space using the inverse of PCA, which is expressed by Equation (13):

(13) $$S_{\mathrm{predict}|1\times m}^{\prime }=Z_{\mathrm{predict}|1\times r}V_{r\times m}^{T}\approx {S^{\prime }}_{1\times m}$$

The obtained $S_{\mathrm{predict}|1\times m}^{\prime }$ is then centered/scaled back to $S_{\mathrm{predict}|1\times m}$. This approach allows us to directly infer the output response for each of the *m* nodes, providing an approximation for the von Mises stress that the input is causing to the model.

#### 2.2.5. ROM Final Architecture Testing

**Figure 4:**
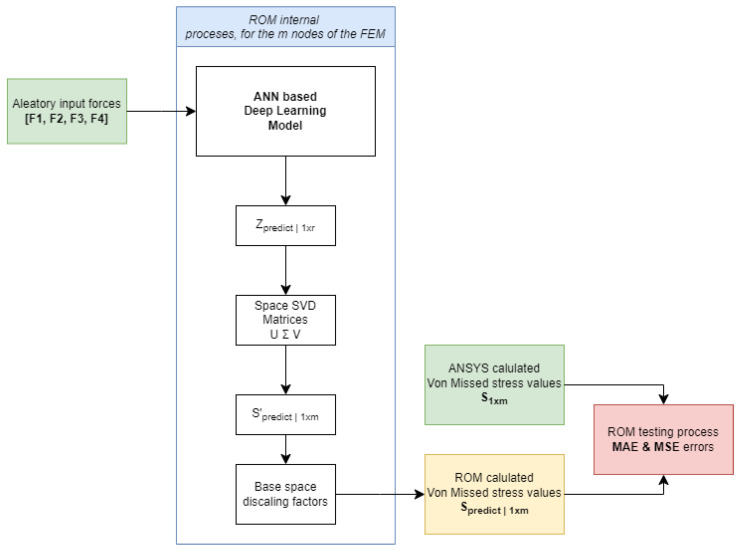
Diagram illustrating the testing process of the ROM architecture, which outputs a final predicted result $S_{\mathrm{predict}|1\times m}$, optimizing computation via base space factors, and compares it to the ANSYS-calculated actual response $S_{1\times m}$.

Here the focus is to compare $S_{\mathrm{predict}|1\times m}$ with a “real” solution directly calculated with ANSYS Mechanical for a general load case combination. Figure 4 represents the internal processes of a ROM using a DL approach for predicting a specific output based on aleatory (random) input forces.

- Input forces: The process begins with four input forces, labeled as $F_{1},F_{2},F_{3},F_{4}$. These inputs are random or aleatory in nature such that $F_{i}\in \left[0,25\right]$ N and serve as the driving factors for the ROM
- DL model: The input forces are fed into an ANN-based DL model. This model processes the inputs to generate an intermediate output, denoted as $Z_{\mathrm{predict}|1\times r}$.
- Space SVD Matrices (**U**, **Σ**, **V**): By applying Equation (13), the intermediate output $Z_{\mathrm{predict}|1\times r}$ is inversed back to $S_{\mathrm{predict}|1\times m}^{\prime }$.
- Discaling process: By inversing the scale process previously executed during the standardization step, finally we obtain $S_{\mathrm{predict}|1\times m}$ as the final output of the ROM.

By this architecture, the ROM implements a structured process, where random input forces are transformed through a DL model and SVD to produce a final predicted output, optimizing the computation through base space factors. The error between the obtained $S_{\mathrm{predict}|1\times m}$ and the real $S_{1\times m}$ is calculated again using the MAE and MSE errors.

## 3. Development of the ROM

Processes required for the creation of the ROM, such as data preprocessing, scaling, and PCA, as well as the internal processes of the ROM shown in Figure 4, are described here.

The code involved in the processes of this section was implemented in the Python language. To ensure the reproducibility of the results, seeds were used in the random processes. A seed is an initial value that pseudo-random number generation algorithms use to produce predictable sequences, allowing experiments to be repeated with identical results. In this case, the seed value was set to 42 for both the dataset splitting (using a Dask module [49] called *dask_ml.model_selection.train_test_split* [49]) and the model training (with a TensorFlow module [50] called *tensorflow.random.set_seed*).

### 3.1. Dataset Processing

The dataset generation process was designed to capture the structural behavior under a comprehensive range of loading scenarios while maintaining computational feasibility. The demonstrator was configured to receive a point load applied at any location on the top platform. To reflect realistic conditions, the load distribution on the four legs of the structure was constrained to produce reaction forces ranging from 0 N to 25 N per leg, discretized in increments of 5 N. This resulted in six possible force values for each leg.

To avoid an exponential growth in combinations, the twelve forces were divided into two groups of four-load combinations, each with six force values (Equations (14) and (15)):

(14) $$\left[F_{1,\;i}\;,\;F_{2,\;i}\;,\;F_{3,\;i}\;,\;F_{4,\;i}\;,\;F_{5,\;i}\;,\;F_{6,\;i}\right]=\left[0.0\;,\;5.0\;,\;10.0\;,\;15.0\;,\;20.0\;,\;25.0\right]\;N$$

(15) $$\left[F_{1,\;i}^{\prime }\;,\;F_{2,\;i}^{\prime }\;,\;F_{3,\;i}^{\prime }\;,\;F_{4,\;i}^{\prime }\;,\;F_{5,\;i}^{\prime }\;,\;F_{6,\;i}^{\prime }\right]=\left[2.5\;,\;7.5\;,\;12.5\;,\;17.5\;,\;22.5\;,\;27.5\right]\;N$$

where i∈{1,2,3,4}, corresponding to a specific leg of the simulated structure.

Considering both groups, the total number of scenarios is defined in Equation (16). This division balances the need for a fine-grained range of forces with the computational constraints associated with FEM simulations. Combining all twelve forces into a single group would have resulted in $12^{4}$ = 20,736 scenarios, an impractical number for FEM calculations. The chosen approach ensures the adequate coverage of plausible loading conditions while maintaining computational efficiency:

(16) $$2\times {\left(6\;\mathrm{load}\;\mathrm{cases}\right)}^{\left(4\;\mathrm{legs}:\;\mathrm{force}\;\mathrm{application}\right)}=2592\;\;\mathrm{scenarios}$$

As detailed in Section 2.1, each combination of loads $F_{1}$, $F_{2}$, $F_{3}$, and $F_{4}$ in the legs of the structure generates a unique stress condition, which is stored in a .txt output file. These files contain the Equivalent von Mises Stress for all 141,100 nodes, as well as their respective IDs and spatial coordinates. These data provide a detailed representation of the structural response for each scenario.

**Figure 5:**
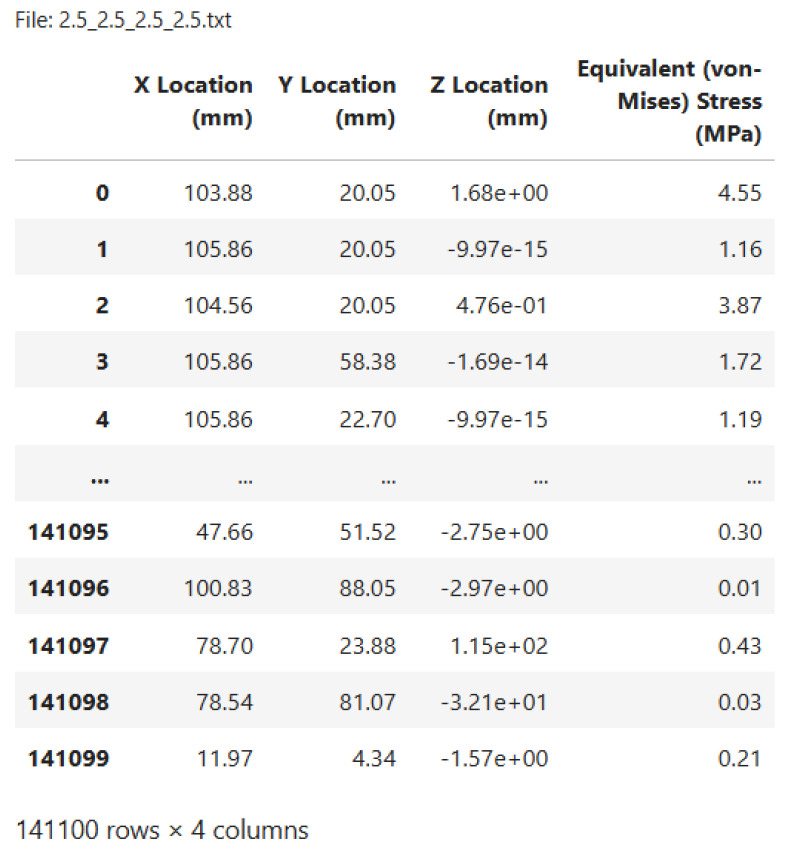
Example of a text file obtained from the FEM simulation, where a load scenario of 2.5 N is equally applied to each force application point in the model. This scenario generates a stress state within the FEM model, accurately representing the real component, allowing the Equivalent von Mises Stress to be determined at each of the 141,100 nodes, along with their spatial locations.

Figure 5 illustrates the format of the output file for a specific load scenario, in which a uniform load of 2.5 N is applied to each force-application point in the model. In them, the position is in units of millimeters (mm) and stress in megapascals (MPa). To streamline analysis, all output files are processed sequentially using a Python [51] script. The script extracts the relevant information and compiles it into a Pandas DataFrame [52], enabling the efficient organization and subsequent analysis of the data.

Nonetheless, in this work, only the Equivalent von Mises Stress is extracted from this file and combined with the load scenario to complete each row of the dataset represented in Table 1. The dataset then consists of input features representing forces applied to different points in the structure and output values representing projection coefficients obtained from the PCA-transformed stress data (Equation (9)).

The next challenge is the large amount of data that need to be processed; forces and stresses were extracted together from the files, resulting in an array with a shape of 1296× 141,104 (141,104 because 4 force columns were added to the ones representing stress at 141,100 nodes). To handle these data efficiently, chunks with a shape of (1296, 1) were defined, ensuring the efficient reading and writing of columns.

The preprocessing step requires performing read and write tasks with the future variables of the dataset. This involves accessing the columns, shaped (1296, 1), of the arrays, which have a shape of (1296, 141,100) when working with stresses or (1296, 141,104) when also working with additional forces. Handling these arrays conventionally would result in RAM overload and would take a significant amount of computation time. Libraries like Dask, which was used in this study, address these problems in a straightforward manner. Some advantages of using Dask over conventional methods include the following:

- Filtering, aggregation, and calculation operations on large datasets can be slow and memory intensive. Dask avoids this by working with chunks.
- It allows working with large datasets that do not fit into memory by processing them sequentially or in parallel.
- Instead of working with a single thread as in the conventional way, Dask’s parallel operations utilize multiple CPU cores, improving performance.
- It allows for deferring the execution of tasks until explicitly calling a method with .compute().

**Figure 6:**
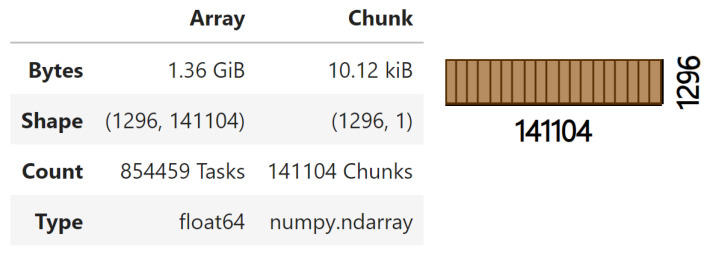
Dask Array visualization showing a total size of 1.36 GiB, shape of (1296, 141,104), and chunks of shape (1296, 1). The array has 854,459 tasks and 141,104 chunks, with each element being a float64 numpy.ndarray. This setup enables the efficient processing of large datasets.

As an example, the total size of an array, such as those used in data preprocessing, is 1.36 GiB, consisting of 854,459 tasks and 141,104 chunks, each with a size of 10.12 KiB. The respective Dask Array divided into chunks is shown in Figure 6.

### 3.2. Scaling and PCA

After preparing the dataset, various partitions were generated to apply PCA and DL algorithms as outlined in Section 2.2.2 and Section 2.2.3. The partition that yielded the best results among all those tested, and which is considered the most orthodox according to the reviewed literature on training regressive numerical models with ANN, is one where the dataset was divided into 70% for training, 20% for validation, and 10% for testing.

To reduce the number of target variables while preserving their interpretability, PCA was applied after scaling the data to a range of 0.1 to 0.9 (see Equation (17)). This scaling minimizes the relative differences between variables, which is crucial, as PCA is sensitive to data scaling. Additionally, the PCA method performs the centering of the variables, giving them a mean of zero. The algorithm performed better by avoiding the extreme values of 0 and 1 during this preprocessing step:

(17) $$\sigma_{i,j}^{\prime }\;=0.1+0.8\times \frac{\sigma_{i,j}-\min\left(\sigma_{i,j}\right)}{\max\left(\sigma_{i,j}\right)-\min\left(\sigma_{i,j}\right)}$$

where we have the following:

- $\sigma_{i,j}$ is the original value of stress (MPa).
- $\sigma_{i,j}^{\prime }$ is the scaled value.
- $\min(\sigma_{i,j})$ is the minimum value of node stress in G matrix.
- $\max(\sigma_{i,j})$ is the maximum value of node stress in G matrix.
- The factor 0.8 is derived from the desired range width (0.9–0.1).
- The addition of 0.1 shifts the entire scale up to start from 0.1.

It is important to highlight that the PCA process is applied only to the training set to avoid contamination with validation and testing data, making these processes more reliable from a scientific standpoint.

**Figure 7:**
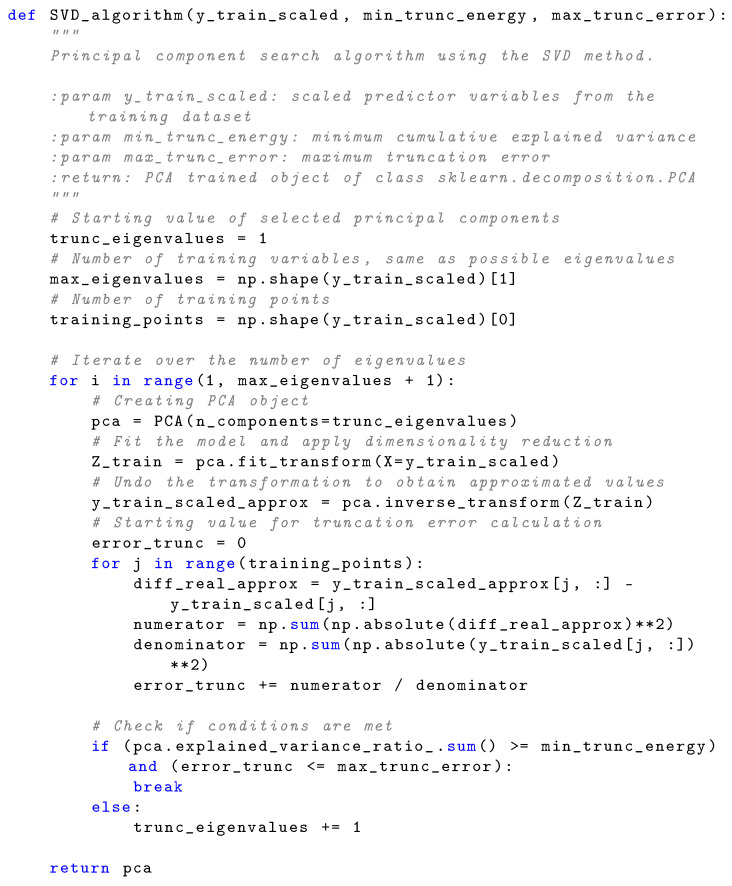
SVD algorithm. It works with the classes from *NumPy* and *sklearn.decomposition.PCA*.

The truncation algorithm in Equation (8), choosing as limits a maximum ${\mathrm{error}}_{\mathrm{trunc}}$ of 1% and a minimum truncation energy (cumulative explained variance) of 95%, led to 125 distinct eigenvalues out of 141,100 possible eigenvalues. Therefore, the SVD was run in a loop, reducing the number of eigenvalues until the above conditions were met. In Figure 7, the code of the SVD algorithm is shown.

**Figure 8:**
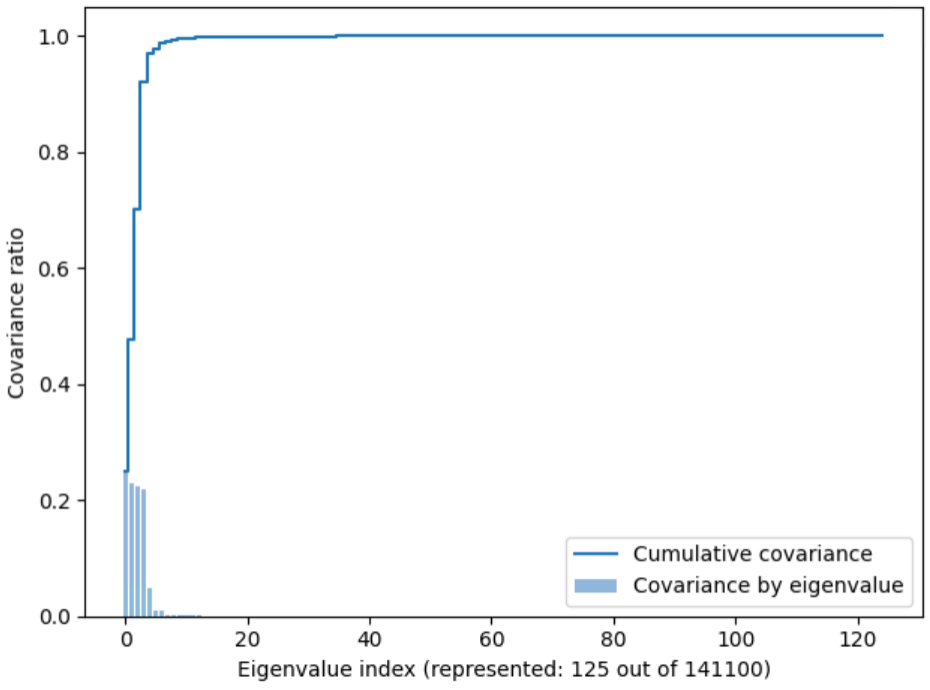
Cumulative variance and variance ratio in function of the number of eigenvalues.

Therefore, the PCA was applied to 70% of the load case combinations in Table 1. Once the principal eigenvalues were identified, this space was used to project the training, validation, and testing matrices onto this reduced space:

1. With 70% of the data (training set), PCA was applied with a 1% truncation. Of the 141,100 possible eigenvalues, we truncated to 125 (see Figure 8). This reduced the numerical space from (1828, 141,100) to (1828, 125).
2. The validation space (20% of the dataset) was projected onto the truncated space. This reduced the numerical space from (500, 141,100) to (500, 125).
3. The testing space (10% of the dataset) was projected onto the truncated space. This reduced the numerical space from (264, 141,100) to (264, 125).
4. In total, we reduced a data space from (2592, 141,100) to (2592,125), which implies a 99% reduction in the data space. This translates from 182 million data points to 0.1 million data points.

### 3.3. Deep Learning Model Training

The DL model used has a sequential neural network by following the methodology described in Section 2.2.3, whose architecture is shown below:

- Dense layer. Units 4. Kernel initializer: Tensor of ones. Activation: ReLU.
  Input shape: (1828, 4)
- Dense layer. Units 80. Activation: ReLU.
- Dense layer. Units 40. Activation: ReLU.
- Dense layer. Units 125.
  Output shape: (1828, 125)

The DL model was trained using the Adam optimizer with a learning rate set to 0.001, and the MSE served as the loss metric. We selected a batch size of 200 and a maximum of 5000 epochs with an early stopping condition of 250 epochs using the EarlyStopping class of Keras.

### 3.4. Hardware and Software Used

The following Table 2 lists the software packages or libraries required for the developed code to run properly, along with their versions and specific purposes.

The hardware used for this study was specifically chosen to meet the computational requirements of both the FEM simulations and the ROM calculations:

- FEM simulations:
  – Processors: two Intel Xeon E5-2630 v4;
  – RAM: 512 GB.
- ROM calculations:
  – Processor: AMD Ryzen 7 5800X (3.8 GHz);
  – RAM: 32 GB DDR4 (2666 MHz).

## 4. Results and Discussion

**Figure 9:**
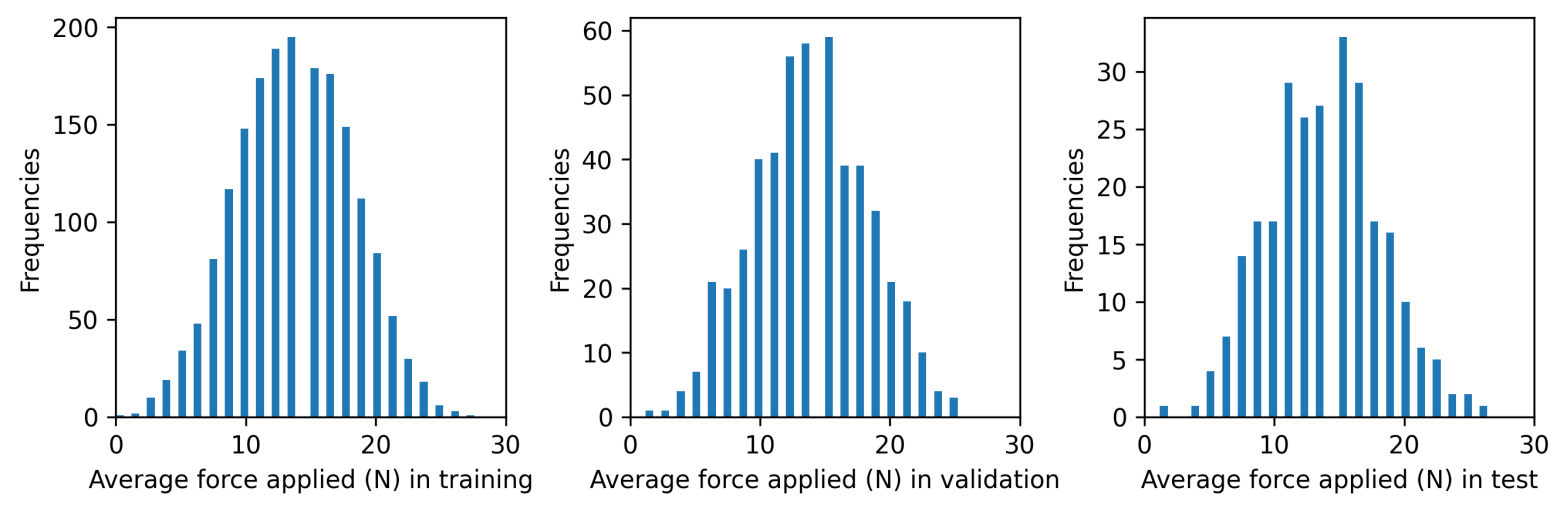
From left to right, distributions of the mean input force values (in Newtons) for the training, validation, and test partitions, respectively.

First, the quality of the train/validation/test split was assessed to ensure that the three subsets maintained the same statistical characteristics as the global distribution. This step was crucial to avoid potential biases that could arise if the data were divided sequentially or non-randomly. As shown in Figure 9, the random splitting ensures that the three subsets share a similar distribution of average input forces. As mentioned in Section 3.1, these values are computed as the mean of each combination of forces applied to the four legs of the structure (see Table 1).

To further quantify the similarity of these distributions, the Kolmogorov–Smirnov (KS) test was applied. The results were as follows:

- train vs. validation: KS Statistic = 0.029, *p*-value = 0.86;
- train vs. test: KS Statistic = 0.042, *p*-value = 0.81;
- validation vs. test: KS Statistic = 0.052, *p*-value = 0.72.

In the KS Test, the null hypothesis ($H_{0}$) states that the two distributions being compared are identical. The *p*-value represents the probability of observing the obtained statistic, or one more extreme, under the assumption that $H_{0}$ is true. If p>0.05, we fail to reject $H_{0}$, meaning the distributions are statistically indistinguishable. Conversely, if p≤0.05, we reject $H_{0}$, indicating that the distributions differ significantly.

In this case, all *p*-values exceed 0.05, confirming that the train, validation, and test subsets are statistically similar to each other. Additionally, the KS statistic, which measures the maximum difference between the cumulative distribution functions (CDFs) of two datasets, is provided here for completeness. The small KS statistic values further corroborate the similarity of the distributions. This ensures that the random splitting preserves the characteristics of the global distribution, making the subsets statistically representative of the full dataset.

**Figure 10:**
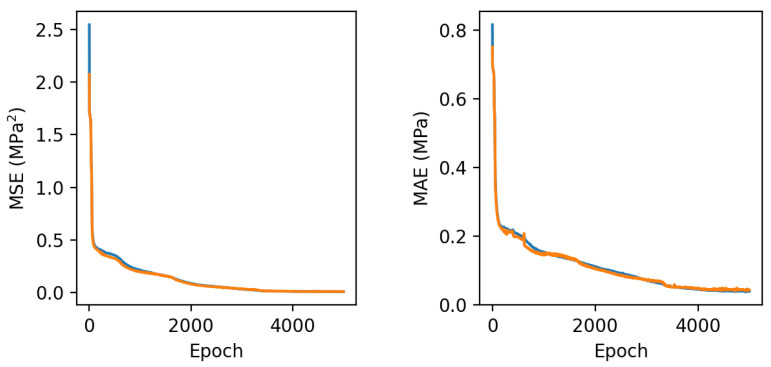
From **left** to **right**, it is shown the models’ metrics evolution of MSE and MAE, respectively, obtained during the processing of the training and validation sets. The blue line corresponds to the training set, and the orange line corresponds to the validation set.

The results presented in Figure 10 reveal the following trends in the network’s performance during training:

- The anticipated asymptotic reduction in the network’s losses as training advances, indicating effective minimization of losses, as well as the MAE;
- Achieving a stable minimum loss by the conclusion of the training phase, signifying completion of the training process;
- Consistent behavior of the model’s losses across both training and validation datasets, suggesting the successful mitigation of overfitting concerns.

**Figure 11:**
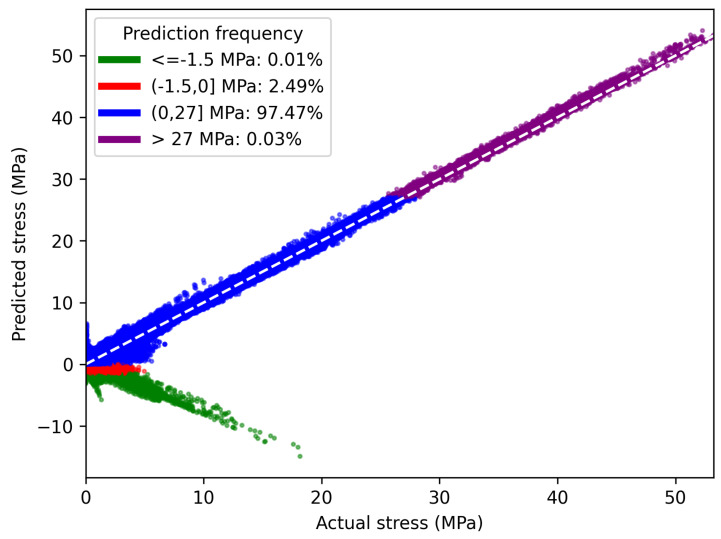
Comparison of the actual values versus the predicted values of stress by the neural network on the test set. Also, a legend is included which reflects the distribution of the predicted values. The dashed white line is the reference for a perfect correlation. Units are in MegaPascals (MPa).

To quantify the degree of correspondence between the predicted stresses of the nodes from the neural network model and the actual simulation values, a correlation plot was used (see Figure 11), which is typical for these cases. This type of plot helps to illustrate how well the predicted values correspond to the actual values, and it provides a visual representation of the correlation between the two variables. The dashed white diagonal line, with a slope of 1, represents the ideal scenario, where the predicted values perfectly match the actual values.

Furthermore, Figure 11 includes a legend to reflect the overall distribution of the predicted data. The main reason for this legend is that the graph contains negative predicted values, and it helps to highlight the fact that most of the negative predictions correspond to values greater than 1.5 MPa, accounting for approximately 2.49% of the total predictions, while negative predictions below this value account for just over 0.01%.

Additionally, about 97.47% of the predictions are positive and less than 27 MPa, while those greater than this value account for only 0.03%. This is to be expected since the high stresses experienced by the studied structure occur at very specific nodes. It should be highlighted that high-stress events are predicted with high accuracy, and they are very important in the potential monitoring applications of this virtual sensor.

Considering these aspects, it is determined that, in general, the scatter plot is very close to the ideal fit. This is further supported by the use of the Pearson correlation coefficient as an evaluation metric, which quantifies the degree of linear alignment between the actual stress values (*X*) and the predicted values (*Y*). The ideal outcome corresponds to a perfect diagonal (Y=X) in the scatter plot, representing an exact match between predictions and actual values.

The scatter plot achieves a MAE value for all events of

(18) $${\mathrm{MAE}}_{\mathrm{test}}=\left(0.04\pm 0.06\right)\;\mathrm{MPa}$$

where the error is the standard error, and there is a Pearson correlation coefficient of 0.998. Both values were calculated with Equations (11) and (12), respectively.

Acceptable error thresholds for Reduced-Order Models (ROMs) are not well defined in the literature. Using the commonly accepted FEM error margin of  5% as a reference, our ROM demonstrates exceptional accuracy as reflected in the calculated ${\mathrm{MAE}}_{\mathrm{test}}=\left(0.04\pm 0.06\right)\;\mathrm{MPa}$ for the instantaneous mechanical stress predictions, evaluated over the entire range of stress values (0 to 5.32 MPa) across the structure. This high precision, combined with significantly faster computational times, offers a valuable benchmark for future ROM applications, despite the scarcity of comparable studies addressing similar mechanical problems and evaluation metrics.

**Figure 12:**
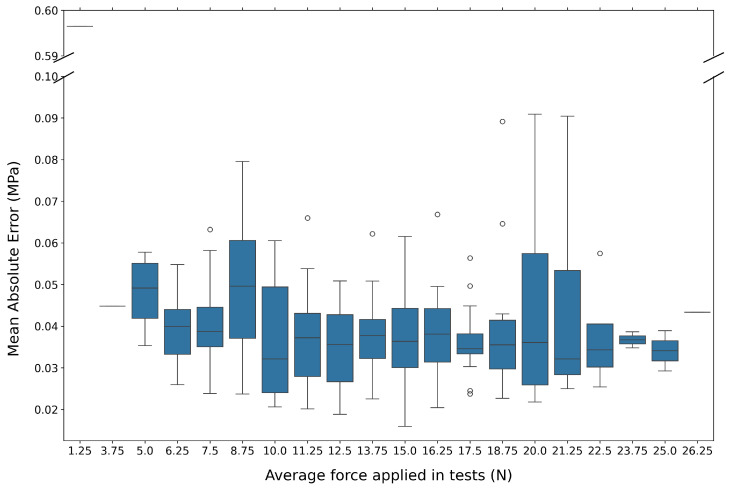
Distribution of the mean absolute error for stress predictions (output of the neural network) for each test case versus the respective average force (input of the neural network). The former is in units of MegaPascals (MPa), and the latter in Newtons (N). The MAE distribution is represented in box plots.

A more tailored approach for this study involves considering the predicted MAE in stresses based on the average force recorded by the sensors on each leg. This representation is shown in Figure 12.

As average force takes discrete values as shown in Figure 9, their corresponding values of MAE are efficiently represented in the form of box plots for each one as can be seen in Figure 12. This plots show the median as a line inside of the box; the third quartile (Q3), which represents the 75th percentile of the data, as the upper limit of the box; and the first quartile (Q1), representing the 25th percentile, as the lower limit of the box. Then, the box itself represents the interquartile range (IQR), which is the range between the first and third quartiles and contains the middle 50% of the data. Also, the plots feature whiskers which extend from the box to the smallest and largest values that are not considered outliers, which are 1.5 times the IQR from the quartiles.

Although a final MAE value for the test set was given in Equation (18), the representation of Figure 9 shows us that the choice of input data for the datasets has a significant influence on the final model. More scattered MAE values are present with more common average force values, which suggests that more cases with extreme average force should be collected when comparing with the distributions in Figure 9. Even so, a slight tendency for greater MAE values with more extreme average force values is observed. This trend is expected because DL models tend to give worse predictions where training data are less prevalent.

Another significant outcome of this project is the comparison of computation times between the FEM simulation of an unknown scenario and its prediction using our ROM model. For this comparison, it is essential to consider the hardware resources utilized for both approaches (see Section 3.4). Using 20 cores for the FEM simulation, the time required to solve the problem was 6 min and 31 s, whereas the ROM prediction took only 0.13 s on the corresponding hardware. This result highlights a substantial advantage and a major milestone for our solution, as the ROM model enables the real-time predictions of the structure’s behavior, effectively functioning as a virtual sensor. Such real-time capability is unattainable with FEM simulations alone.

## 5. Conclusions

This work presents an innovative, robust, and scalable method for creating a high-accuracy, low-computation virtual sensor tailored to mechanical components under linear working conditions. This approach employs a ROM based on ANNs to accurately predict a wide range of output stresses at each node of a mechanical component subjected to a few (but more than one) load inputs, marking a significant advancement in virtual sensing technology. This ANN-based technique effectively handles data complexities beyond the capabilities of conventional computational tools, training the ROM to capture the nodal interrelationships within geometrically intricate components.

Training data were generated through an extensive FEM simulation across multiple scenarios, covering all possible input load combinations and value ranges for each of the four load inputs. The resulting high-dimensional dataset was processed in batches and coordinated with PCA, ensuring scalability for other virtual sensing applications related to mechanical stress in FEM-simulated components with multiple load inputs. This approach will enable the efficient management of large, complex datasets in the future, producing increasingly detailed snapshots as the problem complexity grows. The method processed over 1.36 GiB of information across 854,459 tasks and 141,104 data chunks, significantly improving the processing efficiency, making it feasible to handle datasets that would otherwise be impractical due to memory and computation time limitations.

Model reduction was achieved thanks to a significant simplification of the data space, using PCA and SVD (Section 2.2.1 and Section 3.2). This allowed training the ROM on a normalized projection space, leading to a 99% reduction in the data space. The original data space of 182 million points was reduced to just 0.1 million, representing a substantial gain in computational efficiency without compromising the accuracy needed for the further ANN training process. To support the reproducibility of this research and facilitate the adoption of this methodology, the authors provided code implementing the PCA reduction technique using the scikit-learn library.

Model testing and validation were conducted using a robust sampling method, ensuring that the statistical distributions of stress inputs were consistent across both the training dataset and the testing and validation sets (Section 4). This approach ensured that the model remained consistent, unbiased, and reliable, providing robust and representative results across all phases of the validation process.

The ROM, compared to traditional FEM models, not only gains in computational efficiency but also maintains an acceptable level of accuracy. It shows significant potential for real-time monitoring applications, including stress prediction and predictive maintenance in industrial systems. The ROM error metrics demonstrate an acceptable distribution, with 97.5% of the input values falling within a manageable error range, further proving the model’s reliability for industrial applications.

One of the most significant advantages of the developed ROM is its ability to act as a real-time virtual sensor, replacing traditional FEM models—which require proprietary software such as ANSYS and take seconds to run—with an instantaneous calculation object implemented in an OS environment using Python. This advancement makes the ROM a scalable and viable tool for continuous real-time stress monitoring in structural components, providing immediate feedback within an open-source framework.

Moreover, such models not only enhance predictive capabilities and facilitate proactive maintenance but also enable the real-time regulation of processes affecting the mechanical state of systems. By dynamically adjusting the operating parameters, these models help mitigate the risk of defects, ensuring structural integrity and optimizing system performance.

Furthermore, the achieved results present a clear demonstration of a successful virtual sensor or stress transducer, where the concept of a transducer is key to understanding the system’s functionality. A transducer is a device that converts one form of energy into another—in this case, the mechanical loads (input forces) are converted into stress values distributed across the structural component. The developed ROM acts as a stress transducer by translating four simple load inputs into thousands of stress value predictions throughout the body of a structural element.

The next steps proposed by the authors, which are already underway, involve implementing the ROM in a physical mockup to capture real-time data from mechanical components under force loads. This ROM will function as a virtual sensor, integrated with a ground application and deployed in an edge computing environment. The ROM should be integrated into an open-source (OS) system that collects input from real physical sensors, such as strain gauges, and processes the data to predict the stress distribution across a real component in real-time.

## Figures and Tables

**Table 1:** Dataset structure, illustrating the relationship between input features and output projection coefficients.

Index	Features (Input Forces)				Tags (Output Projection Coefficients)			
	${\mathrm{Foot}}_{1}$	${\mathrm{Foot}}_{2}$	${\mathrm{Foot}}_{3}$	${\mathrm{Foot}}_{4}$	${\mathrm{Coef}}_{1}$	${\mathrm{Coef}}_{2}$	⋯	${\mathrm{Coef}}_{r}$
1	$F_{1,1}$	$F_{1,2}$	$F_{1,3}$	$F_{1,4}$	$z_{1,1}$	$z_{1,2}$	⋯	$z_{1,r}$
2	$F_{1,1}$	$F_{1,2}$	$F_{1,3}$	$F_{2,4}$	$z_{2,1}$	$z_{2,2}$	⋯	$z_{2,r}$
3	$F_{1,1}$	$F_{1,2}$	$F_{1,3}$	$F_{3,4}$	$z_{3,1}$	$z_{3,2}$	⋯	$z_{3,r}$
4	$F_{1,1}$	$F_{1,2}$	$F_{1,3}$	$F_{4,4}$	$z_{4,1}$	$z_{4,2}$	⋯	$z_{4,r}$
5	$F_{1,1}$	$F_{1,2}$	$F_{1,3}$	$F_{5,4}$	$z_{5,1}$	$z_{5,2}$	⋯	$z_{5,r}$
6	$F_{1,1}$	$F_{1,2}$	$F_{1,3}$	$F_{6,4}$	$z_{6,1}$	$z_{6,2}$	⋯	$z_{6,r}$
⋮	⋮	⋮	⋮	⋮	⋮	⋮	⋯	⋮
1296	$F_{6,1}$	$F_{6,2}$	$F_{6,3}$	$F_{6,4}$	$z_{1296,1}$	$z_{1296,2}$	⋯	$z_{1296,r}$
⋮	⋮	⋮	⋮	⋮	⋮	⋮	⋯	⋮
2592	$F_{6,1}^{\prime }$	$F_{6,2}^{\prime }$	$F_{6,3}^{\prime }$	$F_{6,4}^{\prime }$	$z_{2592,1}$	$z_{2592,2}$	⋯	$z_{2592,r}$

**Table 2:** Software packages, versions, and their purposes.

Software Package	Version	Purpose
Dask [49]	2024.5.1	Used for data chunking and parallel computing during data preprocessing.
TensorFlow [50]	2.17.0	Employed for building and training ANNs.
Python [51]	3.12.3	General-purpose programming language used for implementing the workflow.
Pandas [52]	2.2.2	Library for data manipulation and analysis, employed for organizing datasets.
Numpy [53]	1.26.4	Library for numerical computations and array manipulations used throughout.
Scikit-learn [54]	1.5.0	Utilized for PCA and test metrics in ROM.
Keras [55]	3.3.3	High-level API for creating and managing neural network architectures.

## Data Availability

Simulation data will be available upon request by email.
